# Mitochondrial N-formyl methionine peptides contribute to exaggerated neutrophil activation in patients with COVID-19

**DOI:** 10.1080/21505594.2023.2218077

**Published:** 2023-05-29

**Authors:** Runa Kuley, Bhargavi Duvvuri, Jeffrey J. Wallin, Nam Bui, Mary Vic Adona, Nicholas G. O’Connor, Sharon K. Sahi, Ian B. Stanaway, Mark M. Wurfel, Eric D. Morrell, W. Conrad Liles, Pavan K. Bhatraju, Christian Lood

**Affiliations:** a Department of Medicine, Division of Rheumatology, University of Washington, Seattle, WA, USA; b Center for Life Sciences, Mahindra University, Hyderabad, India; c Biomarker Sciences, Gilead Sciences Inc, Foster City, CA, USA; d Department of Medicine, University of Washington, Seattle, WA, USA; e Division of Pulmonary, Critical Care and Sleep Medicine, University of Washington, Seattle, WA, USA; f Sepsis Center of Research Excellence-UW (SCORE-UW), University of Washington, Seattle, WA, USA

**Keywords:** COVID-19, systemic inflammation, neutrophils, clinical biomarkers, N-formyl methionine peptide, mitochondrial peptides

## Abstract

Neutrophil dysregulation is well established in COVID-19. However, factors contributing to neutrophil activation in COVID-19 are not clear. We assessed if N-formyl methionine (fMet) contributes to neutrophil activation in COVID-19. Elevated levels of calprotectin, neutrophil extracellular traps (NETs) and fMet were observed in COVID-19 patients (*n* = 68), particularly in critically ill patients, as compared to HC (*n* = 19, *p* < 0.0001). Of note, the levels of NETs were higher in ICU patients with COVID-19 than in ICU patients without COVID-19 (*p* < 0.05), suggesting a prominent contribution of NETs in COVID-19. Additionally, plasma from COVID-19 patients with mild and moderate/severe symptoms induced in vitro neutrophil activation through fMet/FPR1 (formyl peptide receptor-1) dependent mechanisms (*p* < 0.0001). fMet levels correlated with calprotectin levels validating fMet-mediated neutrophil activation in COVID-19 patients (*r* = 0.60, *p* = 0.0007). Our data indicate that fMet is an important factor contributing to neutrophil activation in COVID-19 disease and may represent a potential target for therapeutic intervention.

## Introduction

The explicit role of host immune responses in the progression of Coronavirus Disease 2019 (COVID-19) disease remains to be fully defined. Dysregulation of both innate and adaptive immune responses in COVID-19 patients has been reported in previous studies [1–4]. In the innate immune response, neutrophils are among the earliest cells in contact with pathogenic agents during airway transmission and mount anti-microbial responses [5]. However, neutrophil responses must be regulated, as hyperactive neutrophil responses have been reported to contribute to the development of severe COVID-19 disease [6,7]. During COVID-19 infection, elevated neutrophil numbers have been reported in pulmonary tissues and blood of critically ill patients with severe respiratory disease, worsening oxygenation, and fatal outcomes [4,8–11]. Variations not only in neutrophil counts but also diversity in neutrophil populations with an abundance of developing pre- and immature neutrophils are observed in severe disease [12,13]. Thus, a prominent role for neutrophils has been implicated in the progression of severe COVID-19 disease due to its altered abundance and phenotype.

Upon neutrophil activation or death, proteins and enzymes stored in the granules or the cytoplasm, such as S100A8/A9 heterodimers (calprotectin), myeloperoxidase (MPO), neutrophil elastase (NE), among others are rapidly released in the circulation [14]. Additionally, the liberation of neutrophil extracellular traps (NETs) through a regulated cell death process termed NETosis is executed by neutrophils for viral inactivation and host protection [15,16]. Elevated levels of both calprotectin and NETs have been found in several infectious and inflammation-associated diseases, such as sepsis, acute respiratory distress syndrome (ARDS), rheumatoid arthritis (RA), systemic sclerosis (SSC), systemic lupus erythematosus (SLE) [17–23]. Similarly, neutrophil activation markers calprotectin and NETs have been reported to be significantly increased in COVID-19. Moreover, a neutrophil activation signature is strongly associated with predictors of critical illness in infected patients [12,24,25], suggesting neutrophils play an eminent role in the perpetuation and exacerbation of COVID-19 disease.

Although several studies point to the role of dysregulated neutrophils in COVID-19, factors contributing to neutrophil-mediated activation in the pathogenesis of COVID-19 pathogenesis remain largely unknown. Many viruses stimulate neutrophils through surface or endosomal Pattern Recognition Receptors (PRRs) resulting in the production of proinflammatory cytokines, chemokines, the release of granular proteins and reactive oxygen species with virucidal effects [26–29]. Among others, damage-associated molecular patterns (DAMPs) such as formyl methionine peptides (fMet) generated by extracellular mitochondrial components are gaining importance as neutrophil activation factors during inflammatory diseases [21,30,31]. fMet is a potent neutrophil chemoattractant acting through the formyl peptide receptor-1 (FPR1) and triggers a variety of neutrophil functions inducing inflammation and tissue damage [32,33]. Due to the potential similarities between COVID-19 and autoimmune disease pathogenesis in the context of the development of inflammation [34], we investigated the role of fMet on neutrophil activation during clinical COVID-19 infections.

In the current study, circulatory profiles of the neutrophil activation biomarker calprotectin, NETs and mitochondrial fMet were assessed in COVID-19 patients with different clinical presentations (mild, moderate/severe, and critically ill receiving invasive mechanical ventilation with COVID-19). Associations of neutrophil biomarkers and fMet levels with several clinical features in COVID-19 patients were investigated, and the role of fMet in neutrophil activation was assessed in COVID-19 patients. Briefly, neutrophil activation markers as well as fMet levels were elevated in COVID-19 patients and associated with various clinical manifestations. Plasma-mediated neutrophil activation through fMet/FPR1-dependent mechanism was evident, suggesting that fMet plays a central role in neutrophil activation and contributes to the pathogenesis of COVID-19.

## Methods

### Patient cohort and ethical statement

Plasma samples from two independent COVID-19 cohorts were analysed in the current study. Among the two cohorts, one was procured from a commercial vendor (MT Group, Los Angeles, CA) and included plasma samples from patients with mild disease (not requiring hospitalization or oxygenation, *n* = 20) and moderate disease (requiring hospitalization and/or oxygenation, *n* = 8), whereas the other cohort consisted of plasma samples from critically ill patients receiving invasive mechanical ventilation with (*n* = 20) or without (*n* = 20) COVID-19 recruited at the University of Washington, Seattle, USA [35] (Table 1). Classification of COVID-19 disease severity was according to the World Health Organization ordinal scale [36]. SARS-CoV-2 infection was confirmed in all patients except for the critically ill COVID-negative group and was confirmed for COVID-19 disease based on real-time polymerase chain reaction (RT-PCR) performed on standard respiratory specimens. In all patients with confirmed COVID-19 disease, plasma was collected once at the time of admission to the inpatient service or the intensive care unit (ICU). The distributions of age, sex, race, ethnicity, patient mortality, clinical characteristics, and treatments received by mild, moderate/severe and critically ill COVID-19 patients are detailed in Table 1. Additionally, demographic and illness severity data of critically ill patients without COVID-19 infection is summarized in Table 1. Healthy controls (*n* = 20) used in this study were recruited from Seattle, WA, and the surrounding area (Table 1). The study was approved by the local Institutional Review Board. Informed written consent was obtained from all study participants according to the Declaration of Helsinki.

**Table 1.** Patient characteristics.

Cohort	COVID-Mild to Moderate	Non-COVID Critically ill	COVID Critically ill	Healthy Controls
Individuals (n)	28	20	20	20
COVID-19 infection	28 (100%)	0 (0%)	20 (100%)	0 (0%)
Age, Mean (SD)	44.1 (15.7)	56.8 (16.8)	57.8 (15.2)	38.6 (12.4)
Male	11 (39%)	15 (75%)	14 (70%)	7 (35%)
Race, White	27 (96%)	9 (45%)	15 (75%)	18 (90%)
Race, Asian	0 (0%)	3 (15%)	4 (20%)	0 (0%)
Race, Black	1 (4%)	4 (20%)	0 (0%)	0 (0%)
Race, Unknown	0 (0%)	4 (20%)	1 (5%)	2 (10%)
Ethnicity, Hispanic/Latino	21 (75%)	3 (15%)	8 (40%)	1 (5%)
BMI, Mean (SD)	30.0 (5.2)	29.6 (6.9)	31.0 (6.0)	28.4 (8.0)
Hospital Mortality	0 (0%)	3 (15%)	8 (40%)	NA
Chronic Kidney Disease	0 (0%)	5 (25%)	0 (0%)	0 (0%)
Diabetes	3 (11%)	7 (35%)	5 (25%)	0 (0%)
Hypertension	ND	13 (65%)	8 (40%)	ND
Mechanically Ventilated Days, Mean (SD)	ND	8.3 (10.3)	20.4 (9.6)	NA
Ventilator Free Days, Man (SD)	ND	19.8 (10.3)	7.6 (9.6)	NA
Berlin Admission ARDS	NA	8 (47%)	17 (85%)	NA
Hospitalized requiring IMV or ECMO	7 (25%)	20 (100%)	20 (100%)	NA
Admission AKI	0 (0%)	11 (55%)	14 (70%)	0 (0%)
Inpatient dialysis	0 (0%)	0 (0%)	3 (15%)	0 (0%)
Steroids	6 (21%)	7 (35%)	8 (40%)	0 (0%)
Remdesivir	8 (29%)	0 (0%)	3 (15%)	0 (0%)
Tocilizumab	2 (7%)	0 (0%)	2 (10%)	0 (0%)

## Neutrophil activation biomarker and fMet assays

Levels of calprotectin in plasma samples were analysed using a commercial ELISA kit according to the manufacturer’s instructions (R&D Systems, Minneapolis MN, USA). Myeloperoxidase (MPO)-DNA and neutrophil elastase (NE)-DNA complexes were assessed to determine NET levels in the plasma samples. MPO-DNA and NE-DNA complexes were measured by ELISA as described previously [20,21,37]. Briefly, anti-MPO antibody (4 μg/ml; MyBioSource, San Diego, CA) and rabbit anti-human NE (4 μg/ml; Calbiochem, San Diego, CA) were used for coating the 96 well ELISA microplates. After blocking, plasma samples (1:50 and 1:10 plasma dilution in 1% BSA in PBS with 2 mM EDTA for MPO-DNA and NE-DNA respectively) were added and incubated overnight at 4°C. Anti-DNA-HRP from the Cell Death Detection ELISA kit (clone MCA-33; Roche) was used as the detection antibody. The reaction was developed with 3,3′,5,5′ tetramethylbenzidine (TMB; BD Biosciences) followed by the addition of 2N sulphuric acid to terminate the TMB reaction. Human N-formyl methionine peptides (fMet) levels were analysed in plasma using a commercial ELISA kit according to the manufacturer’s instructions (My BioSource Inc., San Diego, CA, USA). Absorbance for all ELISA assays was measured at 450 nm with a Synergy plate reader (BioTek). Standards were used as a reference to calculate concentrations of measured analytes in the plasma samples.

## Isolation of neutrophils, *in vitro* activation assays and fMet signalling

Whole blood from healthy individuals was used for neutrophil isolation by using Polymorphprep density gradient (Axis-Shield, Dundee, UK) as described previously [38]. This method results in a yield of >95% neutrophils with >95% viability and approximately 90–95% of neutrophils are viable after neutrophil stimulation with plasma samples [21,39]. Isolated neutrophils were resuspended in serum-free RPMI-1640 medium (Life Technologies, Waltham, MA) for *in vitro* assays. Neutrophils were plated at 2 × 10^5^ cells/well and were pre-incubated for 30 min in the presence or absence of FPR1 inhibitor and cyclosporin H (CsH, 5 μM) followed by addition of stimuli. Plasma from various groups of COVID-19 patients with varying disease severity and healthy controls were used as stimuli. Neutrophils were incubated with stimuli for 2 h followed by neutrophil staining with CD66b (clone G10F5, BioLegend) and CD11b (clone CBRM1/5, BioLegend) monoclonal antibodies. Cell surface neutrophil activation marker levels were assessed by flow cytometry. For flow analysis, the debris were gated out followed by gating of neutrophil based on high side scatter (SSC) and MFI of CD11b and CD66b markers were measured. The data were analysed by FlowJo (Tree Star Inc, Ashland, OR), and the results were presented as CD66b and CD11b mean fluorescent intensity (MFI).

## Statistics

All statistical analyses were performed with Prism software (GraphPad Software). For sample sets with non-Gaussian distribution, non-parametric tests, Mann-Whitney U-test and Spearman’s correlation, were used as applicable. For neutrophil activation assays with plasma stimuli samples, the Mann-Whitney U-test and Wilcoxon’s paired test were performed. A *p* value of < 0.05 was considered significant.

## Results

### Plasma neutrophil activation markers are associated with COVID-19 disease severity

Patients with mild-to-moderate COVID-19 (*n* = 28) were on average 44 years old, mainly women, with the majority of the patients Hispanic (75%). Only 25% were hospitalized with 21% requiring steroids (Table 1). Critically ill patients (*n* = 40) with (*n* = 20) or without (*n* = 20) COVID-19 were on average 57 years old, primarily male (70–75%), with a diverse ethical background with only 30–35% being non-Hispanic White. Critically ill patients experienced a high degree of mortality, with 15% for the non-COVID-19 cohort and 40% for the COVID-19 cohort. Underlying morbidities, including chronic kidney disease, diabetes, and hypertension, were common in the critically ill population. Of note, a majority of critically ill patients with COVID-19 developed ARDS (85%), whereas only 47% of the non-COVID-19 critical ill patients had ARDS (Table 1). Consistently, critically ill patients with COVID-19 had more mechanically ventilated days (20.4) as compared to non-COVID-19 critical ill patients (8.3, Table 1).

To assess the contribution of neutrophils in COVID-19 disease, we measured the neutrophil activation markers calprotectin (S100A8/A9), and NETs (MPO-DNA and NE-DNA complexes) at baseline in plasma samples from COVID-19 patients stratified based on disease severity (mild and moderate/severe disease) and in healthy controls (HCs). Additionally, we assessed the neutrophil activation markers in critically ill COVID-19 patients receiving invasive mechanical ventilation compared to critically ill non-COVID-19 patients.

**Figure 1.**
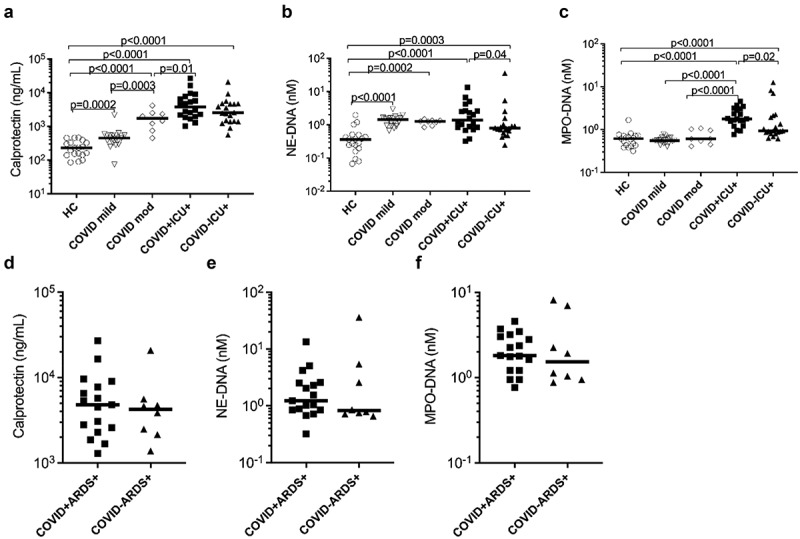
Elevated concentrations of neutrophil activation markers in plasma of COVID-19 patients. Concentrations of neutrophil activation markers (a) calprotectin and (b and c) neutrophil extracellular traps (NETs; measured as neutrophil elastase-DNA complexes, NE-DNA and myeloperoxidase-DNA complexes, MPO-DNA respectively) were measured in stored plasma samples at baseline in COVID-19 patients with disease severity ranging from mild to moderate (mod) to critically ill patients. Comparison of critically ill patients with or without COVID-19 was also performed. (d-f) ICU patients were stratified based on ARDS and assessed for neutrophil activation markers. All assays were analysed by ELISA in plasma samples from COVID-19 patients and healthy controls (HC). Data are represented as dot plot graphs and bars represent the median. Each symbol represents a single subject. Statistics were performed by Mann-Whitney U test.

As compared to the healthy controls, a significant increase in calprotectin was observed in COVID-19 patients with mild (*p* = 0.0002) and moderate/severe disease (*p* < 0.0001). Importantly, the rise of calprotectin levels was more pronounced in patients with moderate/severe disease than those with mild infection (*p* = 0.0003). Consistent with these observations, calprotectin levels were also elevated in COVID-19 ICU patients (*p* < 0.0001) as well as in non-COVID-19 ICU (*p* < 0.0001) patients compared to HC. Although not significant, a trend of increased calprotectin levels was observed in COVID-19 ICU patients compared to non-COVID-19 ICU (*p* = 0.171) patients (Figure 1a). With respect to circulating NETs, only NE-DNA complexes, but not MPO-DNA complexes were elevated in mild (*p* < 0.0001) and moderate/severe disease (*p* = 0.0002) COVID-19 patients compared to HC (Figure 1b,c). A significant increase in both NE-DNA and MPO-DNA complexes levels was observed in COVID-19 ICU patients (*p* < 0.0001) compared to HCs. In contrast to calprotectin levels, no difference in NE-DNA levels was observed irrespective of COVID-19 disease severity (Figure 1b). Furthermore, a modest increase in NE-DNA and MPO-DNA levels was observed in COVID-19 ICU patients compared to non-COVID-19 ICU (*p* = 0.04 and *p* = 0.02 respectively) patients (Figure 1b,c), indicating a more profound increase in NET levels in critically ill patients due to COVID-19 infections as compared to other infections requiring invasive mechanical ventilation. However, of note, in ICU patients with ARDS, there were no differences in neutrophil biomarkers whether COVID-19 or not. Thus, the changes in neutrophil biomarkers likely reflect the severity of the disease, e.g. ARDS, rather than the presence of COVID-19 as such (Figure 1d,f).

## Neutrophil activation marker levels associate with clinical features in COVID-19 patients

**Figure 2.**
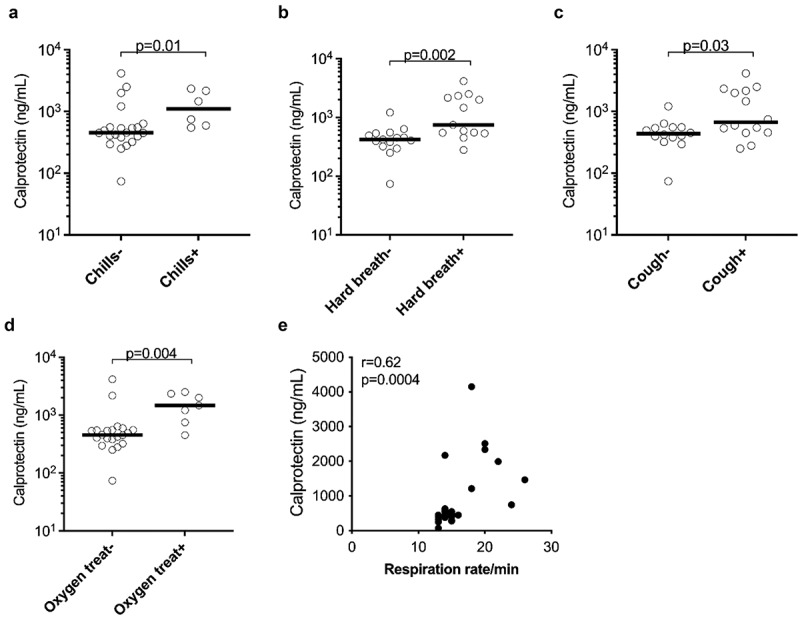
Plasma concentrations of the neutrophil activation marker calprotectin were associated with clinical parameters in COVID-19 patients. Association of plasma calprotectin levels in the presence and absence of (a) chills, (b) cough, (c) hard breathing (hard breath) and (d) oxygen treatment (oxygen treat) in COVID-19 patients. (e) Correlation analysis between calprotectin levels and respiration rate per minute in COVID-19 patients. Clinically well-characterized COVID-19 patients with mild and moderate/severe disease manifestations were used for this analysis. (a-d) Data are represented as dot plot graphs and bars represent the median. Each symbol represents a single subject. Statistics were performed by (a-d) Mann-Whitney U test and (e) Spearman’s correlation test.

To determine whether increased expression of neutrophil markers was associated with clinical features in COVID-19 patients, we assessed the clinical correlations of plasma calprotectin and NET levels in the combined clinically well-characterized mild and moderate/severe COVID-19 patient groups. Higher calprotectin levels were observed in COVID-19 patients clinically presenting with the symptoms of chills (*p* = 0.01) and cough (*p* = 0.03) (Figure 2a,b). In addition, higher levels of calprotectin were also observed in COVID-19 patients with dyspnoea (*p* = 0.002) as well as individuals who developed hypoxaemia (*p* = 0.004) requiring supplemental oxygen therapy (Figure 2c,d). Moreover, elevated levels of calprotectin in COVID-19 patients also correlated with higher respiratory rates (*r* = 0.62, *p* = 0.0004) (Figure 2e). In contrast, NET levels were not elevated in COVID-19 patients with the above-mentioned clinical variables (data not shown). In COVID-19 ICU patients, dialysis use was associated with reduced levels of MPO-DNA (*p* = 0.01, data not shown), and presence of ARDS was associated with elevated calprotectin levels (*p* = 0.04, data not shown). Thus, the neutrophil secretory protein calprotectin was associated with several fever and respiratory disease-related disease phenotypes in mild and moderate/severe COVID-19 patients.

Plasma levels of mitochondrial-derived N-formyl methionine peptides (fMet) are elevated in COVID-19

**Figure 3.**
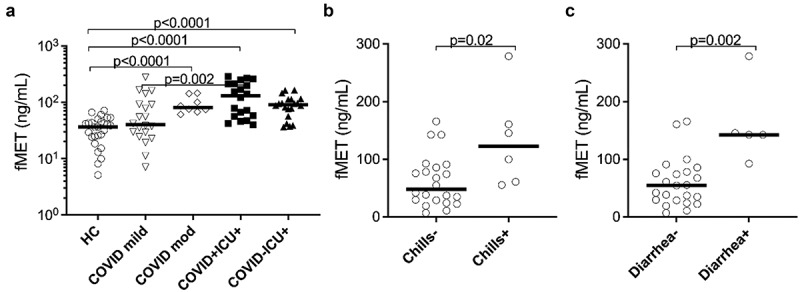
Plasma concentrations of mitochondrial-derived N-formyl methionine peptides (fMet) were elevated in patients with COVID-19 and associated with clinical parameters of the disease. (a) Levels of fMet were measured in plasma samples from mild to critically ill COVID-19 patients and healthy controls (HC) by ELISA. Additionally, fMet levels were analysed in critically ill patients with or without COVID-19. (b and c) Association of plasma fMet levels in the presence and absence of (b) chills and (c) diarrhoea in COVID-19 patients. Clinically well-characterized COVID-19 patients with mild and moderate/severe disease manifestations were used for associations between plasma fMet levels and clinical variables of disease. Data are represented as dot plot graphs and bars represent the median. Each symbol represents a single subject. Statistics were performed by the Mann-Whitney U test.

As shown in Figure 3a, total fMet levels were elevated in COVID-19 patients with moderate/severe disease (*p* < 0.0001) and in ICU patients with (*p* < 0.0001) or without COVID-19 infection (*p* < 0.0001) compared to HC. In contrast, no significant changes in fMet levels were observed in COVID-19 patients with mild symptoms (*p* = 0.1416) relative to HC. Interestingly, fMet levels were significantly higher in COVID-19 ICU patients compared to patients with mild COVID-19 manifestation (*p* = 0.002). Similar to calprotectin levels, a trend of increased fMet levels was observed in COVID-19 ICU patients compared to non-COVID-19 ICU (*p* = 0.157) patients. Additionally, higher fMet levels from mild and moderate/severe COVID-19 patient groups were associated with clinical parameters of fever such as chills (*p* = 0.02) (Figure 3b) and gastrointestinal symptom diarrhoea (*p* = 0.002) (Figure 3c).

fMet induces neutrophil activation via FPR1 receptor and associates with neutrophil activation marker in COVID-19

Mitochondrial-derived fMet is the principal cognate ligand of the FPR1 receptors and can induce neutrophil effector functions [40]. We investigated whether elevated levels of fMet present in COVID-19 patient plasma could promote neutrophil activation through FPR1. To this extent, we performed de novo neutrophil activation assays, where neutrophils isolated from healthy individuals were incubated with plasma samples from COVID-19 patients and HC. Plasma-mediated neutrophil activation was assessed by analysing cell surface expression of neutrophil activation markers CD66b and CD11b by flow cytometry.

**Figure 4.**
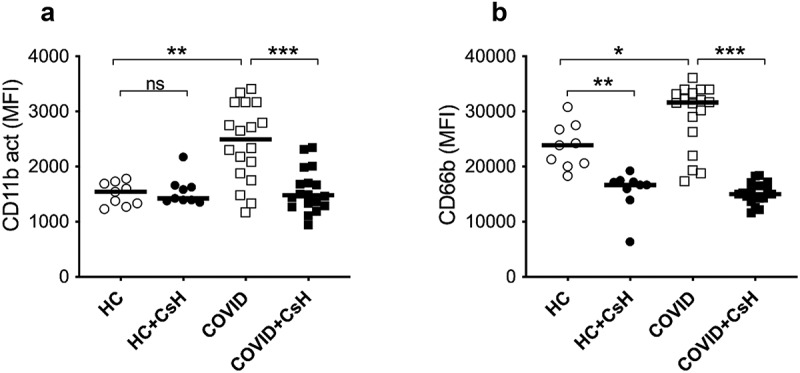
Mitochondrial fMet contributes to neutrophil activation by signalling through FPR1 receptors. Neutrophil activation upon incubation for 2 hours with plasma from HC and COVID-19 patient samples with mild and moderate disease manifestation. Neutrophils were pre-incubated in the presence or absence of FPR1 antagonist Cyclosporine H (CsH) and neutrophil activation markers (a) CD11b and (b) CD66b were assessed by flow cytometry. Data is represented as dot plot graphs indicating the MFI of CD11b and CD66b and bars represent the median. Each symbol represents a single subject. Statistics were performed by Mann-Whitney U test and Wilcoxon test. NS: Non-significant.

Upon incubation of neutrophils with plasma samples from COVID-19 patients having either mild or moderate/severe disease phenotypes, neutrophils were strongly activated, as demonstrated by increased neutrophil cell surface expression of CD11b (*p* = 0.0015) and CD66b (*p* = 0.017) as compared to neutrophils exposed to plasma from HC (Figure 4a,b). For this analysis, the COVID-19 mild and moderate/severe groups were combined as no differences between these groups of COVID-19 patients were observed with regard to their capacity to induce in vitro neutrophil activation (data not shown). A significant decrease in neutrophil expression of CD11b (*p* < 0.0001) and CD66b (*p* < 0.0001) was evident in the presence of FPR1 inhibitor and cyclosporine H (CsH), suggesting an FPR1-dependent neutrophil activation. Unexpectedly, we also observed reduced neutrophil CD66b expression in HC samples in the presence of CsH (Figure 4b). It should be noted that FPR1 can shift between active and inactive conformations, similar to other G-protein coupled receptors, with some spontaneous signalling occurring at baseline. Cyclosporin H locks FPR1 into its inactive form, preventing even basal signalling from occurring through this receptor. Thus, it is likely that the reduction in CD66b expression observed in HC is driven through the ability of Cyclosporin H to prevent spontaneous FPR1 signalling.

**Figure 5.**
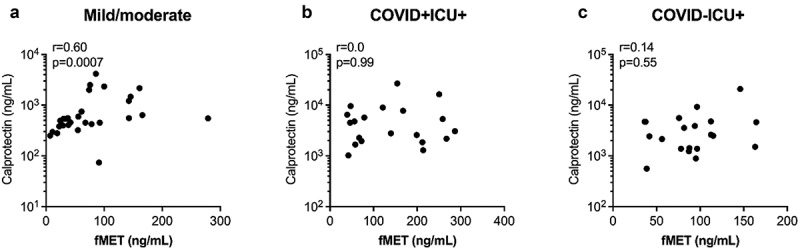
Plasma concentrations of fMet are associated with calprotectin in COVID-19 patients. Levels of fMet and calprotectin were assessed by ELISA in patients with various disease subgroups. Correlation analysis between calprotectin and fMet from (a) mild and moderate and (b) critically ill patients with COVID and (c) critically ill patients without COVID are shown. Each symbol represents a single subject. Statistics were determined by Spearman’s correlation test.

Because plasma-mediated neutrophil activation through fMet/FPR1 signalling was observed in COVID-19 patients, we next assessed the association of neutrophil activation marker calprotectin with fMet levels. In the combined mild and moderate/severe symptoms group of COVID-19 patients, levels of calprotectin correlated significantly with fMet levels (*r* = 0.60, *p* = 0.0007) (Figure 5a), an observation consistent with fMet/FPR1-mediated neutrophil activation from this subgroup of COVID-19 patients (Figure 4). No correlation was found between levels of calprotectin and fMet in ICU patients in the presence (*r* = 0.003, *p* = 0.99) or absence (*r* = 0.14, *p* = 0.55) of COVID-19 (Figure 5b,c). Thus, mitochondrial-derived fMet may be a crucial factor contributing to neutrophil-mediated inflammation in COVID-19 in an FPR1-dependent manner.

## Discussion

Neutrophils play fundamental roles in host defence as innate immune cells via several mechanisms, including NET formation. Although beneficial in eradicating invading pathogens, exaggerated neutrophil activation may result in inflammation and end-organ damage, as seen in several inflammatory conditions, including rheumatoid arthritis (RA) and systemic lupus erythematosus (SLE) [20,22]. The current study provides an overview of expression profiles of neutrophil activation markers in COVID-19 patients, with clinical presentations ranging from mild-to-moderate symptoms to critically ill patients. Our findings indicate that the circulating markers of neutrophil activation are elevated in critically ill COVID-19 patients and have a high discriminatory value. Similar findings of prominent neutrophil activation have also been recognized in the disease pathogenesis of COVID-19, with neutrophil activation reflecting a severe disease, often predicting worse clinical outcome [4,8–12,24]. As neutrophils play a critical role in the development of severe disease and subsequent organ damage, there is a compelling need to decipher mechanisms associated with exaggerated neutrophil activation in response to COVID-19. In this study, we demonstrated the involvement of mitochondrial N-formyl methionine (fMet) mediated neutrophil activation via the FPR1 pathway, indicating a potential therapeutic pathway for intervention to mitigate neutrophil-mediated inflammation in COVID-19 disease. Targeting this pathway, similar to what is seen in animal models [41–44], is anticipated to result in reduced neutrophil-mediated inflammation and damage in COVID-19-related lung injury.

In the current study, we used plasma samples from the patients unlike previous studies where serum samples were primarily used to assess neutrophil activation markers [45–49]. Physiological levels of biomarkers are reflected better in plasma than serum samples as plasma samples are more stable. Additionally, the serum processing steps might affect the physiological measurements of certain markers, such as NETs, where artificial NETs are formed during the processing steps [50,51]. Thus, our comprehensive study provides a credible proof of the contribution of neutrophil activation in COVID-19 diseases and the involvement of factors, such as extracellular mitochondrial proteins fMet in activation of neutrophils. Additionally, COVID-19 infection has become the dominant cause of ARDS and activation of neutrophils plays a key role in it [52]. Ours is one of the foremost studies comparing neutrophil activation markers in ARDS patients induced with or without COVID-19 infection. Our study shows an increased neutrophil activation signature implicating exaggerated neutrophil activation due to COVID-19 infection. Both viral-related effects and inflammatory substances derived from host cells can cause the pathogenesis of ARDS. Patients with ARDS developed due to COVID-19 infection have high lung compliance, and their dependence on mechanical ventilation is longer than that of non-COVID-19 ARDS [53–55]. This could be attributed to robust inflammatory reactions by neutrophils causing prolonged lung and systemic inflammation and aggravation of ARDS in COVID-19 infected patients.

During infections, neutrophils egress from the bone marrow into the circulation and enter the sites of infection to clear pathogens through phagocytosis and oxidative burst mechanisms [26,56]. However, increased circulating activated neutrophils, have been shown to be an independent predictor for disease severity and death in COVID-19 [25]. This could be due to potential overdrive of neutrophils against the virus by secretion of several inflammatory components and alarmins extracellularly in circulation-like alpha-defensins (DEFA1), calprotectin, myeloperoxidase (MPO), neutrophil elastase (NE), chemokines, cytokines (IL-6, IL-8) etc. [14,26,45,57]. Similar to our study, calprotectin has been consistently shown to be significantly upregulated in COVID-19 infected patients with higher levels in patients with severe disease [58]. In particular, our study showed a strong relationship between calprotection levels and severe respiratory disease in COVID-19 consistent with previous studies [58]. Moreover, calprotectin levels were also associated with thrombotic events in COVID-19 patients probably due to engagement of calprotectin with RAGE and TLR4 by activation of these innate immune sensors [47,58–60]. In adults, increased levels of calprotectin were also observed in children suffering with multisystem inflammatory syndrome (MIS-C) after COVID-19 infection [61]. More importantly, calprotectin was a significant determinant if ICU admission and invasive mechanical ventilation were required at any point during hospitalization by COVID-19 patients [47,49,62]. These observations highlight the pathological significance of calprotectin levels with the potential to predict the outcome of severe COVID-19 disease in both adults and children, which deserves to be further explored.

Neutrophils are the first line of defence against pathogens and the role of neutrophils to immobilize virus particles as an antimicrobial strategy during infections is well established [7,63,64]. However, virus-induced NETs have the potential to both control the pathogen and damage the host acting as a pathogenic mediator during inflammatory conditions [15]. Our study found an overall upregulation of NETs in critically ill COVID-19 patients consistent with previous work [24,46,65,66]. Among NETs, NE-DNA complexes were not necessarily specific to severe disease manifestations, unlike the MPO-DNA complexes, which are upregulated only in critically ill patients and could be used as a specific biomarker to predict the development of severe disease in COVID-19 infected patients. Our data are consistent with recent work, which demonstrated that neutrophils from individuals with COVID-19 are primed to undergo enhanced NET formation [67]. The reason for the enhanced capacity to induce NET formation in COVID-19 ARDS is uncertain, with prior work conflicting on whether soluble components, including inflammatory cytokines, may act as prime neutrophils [46]. Although serum from patients with mild and moderate COVID-19 induced NET formation in neutrophils from healthy individuals [46], plasma from critically ill patients with COVID-19 ARDS surprisingly failed to induce NET formation [67]. It is noteworthy that other than difference in disease severity, the first study used serum and not plasma for priming neutrophils. We and others have published on the artificial activation of neutrophils upon coagulation of serum, with elevated levels of calprotectin and NETs observed in serum as compared to plasma [20]. Further, platelet activation, as occurs upon coagulation, is known to promote NET formation, both directly through binding of the platelet to the neutrophil and indirectly by platelet-derived soluble mediators [68,69]. It is thus possible that coagulation may contribute to generating the NET-inducing capacity of serum. However, it is also possible that different mechanisms contribute to neutrophil activation and NET formation in mild/moderate vs severe COVID-19, which will have to be addressed in future studies.

Neutrophils express a plethora of pattern recognition receptors, being able to respond to a wide range of danger-associated molecular pattern. Thus, plasma (or serum) from patients with inflammatory conditions commonly are able to induce neutrophil activation in vitro [20,21,31]. Consistently, plasma from patients with mild or moderate COVID-19 induced neutrophil activation, and we identified fMet as the chief contributor to this process. fMet in humans is uniquely expressed in mitochondria, acting as a danger-associated molecular pattern upon release by activated platelets, cell death, and/or tissue damage, to prime monocytes and neutrophils for chemotaxis and infiltration into tissue via FPR1. Of note, levels of fMet were elevated in patients with COVID-19, particularly in critically ill patients. Elevated levels of fMet were recently described in non-COVID-19 critically ill patients, associated with a metabolic shift, and heightened mortality [70], as well as in patients with other inflammatory conditions, including vasculitis, SSC, and RA, wherein fMet levels contributed to neutrophil activation [21,30,31]. However, in COVID-19 patients, levels of fMet only correlated with levels of neutrophil activation markers (calprotectin) in patients with mild-to-moderate disease, whereas for critically ill patients, no such correlations were observed. As such, fMet may be an important contributor to neutrophil activation in mild disease, whereas other mechanisms may contribute to neutrophil activation in critically ill patients.

Given its prominent role in neutrophil recruitment and activation, FPR1 has been investigated in several inflammatory lung injury models, with FPR1 knock-out ameliorating disease in several models, including acute endotoxin-induced lung injury, DNBS-mediated colitis, as well as models of chronic obstructive pulmonary disease and pulmonary fibrosis [41–44]. Thus, FPR1 may be a novel therapeutic target for COVID-19-mediated inflammation and lung disease.

In summary, our data highlight the clinical value of measuring neutrophil-derived activation markers in COVID-19, and more importantly, identifying a novel potential therapeutic target, FPR1, to regulate neutrophil-mediated inflammation and end-organ damage in COVID-19.

## Data Availability

The datasets generated during and/or analysed during the current study are available from the corresponding author on reasonable request.
